# Editorial: Microvascular dysfunction and organ failure during cardiac surgery

**DOI:** 10.3389/fmed.2023.1231464

**Published:** 2023-06-12

**Authors:** Charissa E. van den Brom, Carolien S. E. Bulte

**Affiliations:** ^1^Department of Anesthesiology, Amsterdam UMC, VU University, Amsterdam, Netherlands; ^2^Department of Intensive Care Medicine, Amsterdam UMC, University of Amsterdam, Amsterdam, Netherlands

**Keywords:** endothelium, microcirculation, cardiopulmonary bypass, hypoxia, vascular dysfunction, techniques

Since the start of cardiac surgery with cardiopulmonary bypass in the 1950s, considerable research has been performed to reduce the detrimental effects of cardiac surgery. Developments such as minimally invasive cardiac surgery and off-pump coronary bypass, but also optimization of cardioplegic, priming and anesthetic strategies have contributed to the overall reduction in patient morbidity and mortality. Despite these advances, the microcirculation remains a black box during cardiac surgery. It is however clear that microvascular dysfunction is considered a significant contributor to organ failure.

The vascular endothelium holds a central position in the regulation of vascular tone, inflammation, fluid transport, and coagulation. The activation of the endothelium by inflammatory mediators triggers a pro-inflammatory as well as a pro-coagulant state. These changes result in amongst others shedding of the endothelial glycocalyx layer, increased vascular permeability, interstitial fluid accumulation and disturbed microcirculatory perfusion (1). As microcirculatory perfusion is essential for tissue delivery of oxygen and nutrients, microcirculatory perfusion disturbances leads to hypoxia and organ dysfunction. The pathophysiology is however complex and not thoroughly understood, impeding its translation into clinical practice.

Therefore, we, as co-guest editors, are pleased to present the Research Topic collection “*Microvascular dysfunction and organ failure during cardiac surgery*” in Frontiers in Medicine. We aimed to highlight the vascular endothelium as a relevant player in the development of organ dysfunction in patients undergoing cardiac surgery using cardiopulmonary bypass. The collection starts with an extensive review by Kant et al. emphasizing the broad aspects of microvascular dysfunction during cardiac surgery, such as dysregulated vasoactive responses, complement activation, inflammation and ischemia and reperfusion injury. Interestingly, the authors zoomed in on critical organs such as the kidneys, brain and lungs and discussed the commonly neglected organ-specific microvascular consequences of cardiac surgery. The consequences of cardiac surgery on one of these critical organs, the kidney, was studied by Fan et al.. It is already known that cardiac surgery associated-acute kidney injury (CS-AKI) occurs in up to 30% of the patients. In this retrospective cohort study, the authors reported that almost 20% of the 564 patients with CS-AKI progressed to chronic kidney disease within 90 days. Interestingly, it was found that risk factors for the progression to chronic kidney disease were vascular-related complications, such as hypertension, coronary artery disease and congestive heart failure. These data emphasize the need for specific techniques to assess (the consequences of) microvascular dysfunction in an earlier stage and to develop new treatment strategies protecting the microcirculation.

Currently, multiple techniques exist to assess the microcirculation, with handheld video microscopy standing out as the most advanced approach at a patient's bedside (2). It is however important to understand that these techniques come with certain limitations, such as accessibility, observer bias and non-automatic analysis. In this Research Topic, two monitoring techniques were proposed; measurements of gastrointestinal blood flow and cutaneous mitochondrial oxygen tension (mitoPO_2_). Point-of-care ultrasound was used to measure blood flow in the superior mesenteric artery, the largest blood supply to the small intestine. Cardiac surgery-induced increased intestinal microcirculatory resistance associated with higher lactate levels post-cardiac surgery and longer ICU stay (Zhou, He, Wang, et al.) and predicted prolonged mechanical ventilation time (Zhou, He, Cui, et al.). Whether alterations in gastrointestinal blood flow are related to microcirculatory alterations in critically affected organs needs to be determined. Interestingly, Harms et al. compared mitoPO_2_ to tissue oxygenation (StO_2_) as measured by near-infrared spectroscopy. Mitochondria are the end-users of oxygen and mitoPO_2_ reflects the local balance between oxygen supply and oxygen consumption within the cell. The data suggest that mitoPO_2_ is an earlier and more sensitive indicator of tissue hypoxia compared to StO_2_. Remarkably, these pilot data showed that patients who developed AKI had a significantly longer time below the mitoPO_2_ threshold of 20 mmHg. It is important to realize that for understanding and interpretation of these measurements, clinical trials need to be conducted.

In summary, the studies presented in this Research Topic contribute to a greater awareness of microvascular dysfunction during cardiac surgery and emphasize the current need for techniques to determine microvascular dysfunction in critical organs in order to develop strategies to further improve cardiothoracic patient care. We thank the authors for their original contributions.

## Author contributions

Both authors listed have made a substantial, direct, and intellectual contribution to the work and approved it for publication.
